# The After Diagnosis Head and Neck cancer-specific Patient Concerns Inventory (HaNC-AD) as a pre-treatment preparation aid during the COVID-19 pandemic

**DOI:** 10.1007/s00405-020-05995-9

**Published:** 2020-04-27

**Authors:** Anastasios Kanatas, S. N. Rogers

**Affiliations:** 1 Leeds Teaching Hospitals, St James Institute of Oncology, Leeds Dental Institute, Leeds General Infirmary, Leeds, LS1 3EX UK; 2 University Hospital Aintree, University of Liverpool, Liverpool, UK

**Keywords:** Patient concerns inventory, Head and neck cancer, SARS-CoV-2, Coronavirus

## Abstract

The coronavirus disease 2019 pandemic has resulted in new challenges for clinicians, head and neck cancer (HNC) patients and carers. There is evidence that the current crisis is affecting the management of HNC patients. Most healthcare systems have introduced remote consultations to decrease the risk of coronavirus infection to patients, carers and clinicians. At present, HNC patients may be anxious and due to logistical issues, may not be adequately prepared for their treatment. To ensure that patients have a thorough understanding of their treatment and expected outcome during the current COVID-19 crisis there may be merit in the use of the HaNC-AD PCI.

The coronavirus disease 2019 (COVID-19) pandemic resulted in challenges for clinicians, head and neck cancer (HNC) patients and carers that were never previously encountered [1]. Some of these challenges are related to social distancing and reduced clinical contact. There is evidence that COVID-19 affects the management of patients with cancer, [2] but at present we have no effective strategy to ensure that cancer patients receive adequate information prior to their treatment. Currently there is reduced clinic contact and this makes it more difficult to share information about cause, stage and treatment outcomes with patients and carers. When diagnosed with cancer there is potentially a lot of information to take in. Over the years, clinical teams perfected ways of ensuring that every patient is adequately prepared to face their treatment. During this crisis, surgical capacity is being significantly reduced to provide critical care facilities as the incidence of COVID-19 increases. Work has commenced to prioritise surgical cases. This will have a detrimental impact on HNC treatment. Clinicians treating HNC patients have to balance the risk in terms of potential exposure to COVID-19 during the treatment, inpatient peri-operative care or outpatient adjuvant treatment. As a consequence of this, patients may have to be prioritised faster and reach the definitive treatment day significantly quicker after receiving their bad news of their diagnosis. Also, a proportion of patients will be significantly delayed and this will add to uncertainty about their care and possible outcome. The issue related to a diagnosis of HNC needs to be discussed carefully and sensitively. A prompt list can help patients and their carers raise any issues which for many reasons under the current crisis, fail to disclose before treatment.

The COVID-19 pandemic has introduced extra worries to patients with a diagnosis of head and neck cancer. There is the risk that during the current crisis, some patients may not be prepared emotionally or psychologically to receive life-changing treatment. This is exacerbated by the required self-isolation guidance that may have an impact on the availability of specific members of the clinical team such as specialist nurses, speech and language therapist or dieticians. During this crisis, there may be merit in the use of the HaNC-AD PCI [3] as a preparation for treatment aid after a diagnosis of head and neck cancer and this can be done remotely during a telephone consultation.

In the head and neck oncology setting, the HaNC-AD PCI [4] encompasses a comprehensive item prompt list covering several broad domains pertinent to patients with a diagnosis of cancer to the head and neck (“Appendix 1”). It is based on the same principle as the PCI-HNC [5] and in a recent systematic review [6] and content comparison of unmet needs self-report measures favoured the PCI over 13 other tools. This is a patient-reported tool, specifically designed for head and neck cancer patients, completed by patients following diagnosis prior to treatment that can facilitate discussion with the wider multi-professional team. It is designed to help evoke patient concerns and may be completed by the patient at home after the diagnosis. This can then be fully evaluated by the clinical team via a follow-up telephone consultation prior to treatment. It is very hard to get the amount of information right and it needs to be tailored for each individual patient especially during this crisis. The information needs to be at the 'right' time for the patient and this can be just after the diagnosis.

In our practice following a remote or a face-to-face appointment to inform the patient of head and neck cancer diagnosis, we give a paper or an electronic copy to the patient. Then a member of the treating team contacts the patient over the phone 3 days later and discusses any identified issues. The HaNC-AD PCI gives the opportunity for the patient to consider and reflect on aspects of their care within a relatively short time frame, allows information exchange remotely and gives them the opportunity to assimilate, understand the treatment and provide informed consent. This simple tool can be given to patients and then be evaluated by the clinical nurse specialist prior to the definitive treatment.

Our preliminary experience is that the HaNC-AD PCI may provide a very useful tool prior to treatment delivery during this crisis, with information delivered remotely by the clinical team.

However, more clinical evidence is needed to ensure that such a strategy is optimal for head and neck cancer care.
